# Larvicidal Activities of *Juniperus chinensis* var. *kaizuka* Leaf Essential Oil and Its Constituents Against Dengue Vector Mosquitoes, *Aedes aegypti* and *Ae. albopictus*

**DOI:** 10.3390/plants14213321

**Published:** 2025-10-30

**Authors:** Ji-Yun Chang, Kun-Hsien Tsai, Yu-Mei Huang, Yu-Yi Chang, Chong-Syuan Huang, Yu-Tung Ho, Sheng-Yang Wang, Mei-Ling Chang, Hui-Ting Chang

**Affiliations:** 1 School of Forestry and Resource Conservation, National Taiwan University, Taipei 10617, Taiwan; r10625005@ntu.edu.tw (J.-Y.C.); r10625006@ntu.edu.tw (Y.-Y.C.); r12625004@ntu.edu.tw (C.-S.H.); r10625032@ntu.edu.tw (Y.-T.H.); 2 Department of Public Health, National Taiwan University, Taipei 10055, Taiwan; kunhtsai@ntu.edu.tw; 3 Department of Entomology, National Taiwan University, Taipei 10617, Taiwan; 4 Institute of Environmental and Occupational Health Sciences, National Taiwan University, Taipei 10055, Taiwan; d95841007@ntu.edu.tw; 5 Department of Forestry, National Chung Hsing University, Taichung 40227, Taiwan; taiwanfir@dragon.nchu.edu.tw; 6 Special Crop and Metabolome Discipline Cluster, Academy Circle Economy, National Chung Hsing University, Taichung 40227, Taiwan; 7 Department of Food Science, Nutrition, and Nutraceutical Biotechnology, Shih Chien University, Taipei 10462, Taiwan

**Keywords:** *Juniperus chinensis* var. *kaizuka*, brine shrimp lethality activity, mosquito larvicidal activity, *Aedes aegypti*, *Aedes albopictus*

## Abstract

*Juniperus* is one of the vital genera of the Cupressaceae family; many *Juniperus* species (juniper) have served as traditional folk medicines. The aims of this study are to analyze its chemical composition and to evaluate the mosquito larvicidal activity of leaf essential oil and its constituents. The constituents of leaf essential oil were analyzed by GC-MS. Leaf essential oil is mainly composed of hydrocarbon monoterpenes and, secondly, oxygenated monoterpenes. Leaf essential oil exhibited good brine shrimp lethality activity, which is highly correlated with larvicidal activity, with the LC_50_ of 49.89 μg/mL. Leaf essential oil showed a strong mosquito larvicidal activity against two Dengue vector mosquitoes, *Aedes aegypti* and *Ae. albopictus*, the LC_50_ values for both species were lower than 50 μg/mL. Among the major constituents of leaf essential oil, compounds limonene, sabinene, and β-myrcene also exhibited a significant larvicidal effect. Through these investigations, it is expected that leaf essential oil from *J. chinensis* var. *kaizuka* and its constituents are of potential use as environmental control chemicals against Dengue vector mosquitoes.

## 1. Introduction

*Juniperus* spp. (junipers), which includes more than 60 species, are evergreen trees and widely distributed throughout the Northern Hemisphere, including temperate and subtropical regions. Junipers are found in Europe, Asia, North America, and even in alpine mountainous areas of the tropics, such as Africa. Research showed that berry extracts of *Juniperus communis* during the first and second year of maturity also displayed antibacterial and antioxidant activities [1]. Hrytsyna et al. examined the chemical constituents and antioxidant activity of juniper cone berries collected from different populations based on a cluster analysis. Tang et al. analyzed the phenol in juniper and hops, a total of 148 phenolic compounds were tentatively identified in juniper and hops (*Humulus lupulus*), among which phenolic acids (including hydroxybenzoic acids, hydroxycinnamic acids and hydroxyphenylpropanoic acids) and flavonoids (mainly anthocyanins, flavones, flavonols, and isoflavonoids) were the main polyphenols by using LC-ESI-QTOF/MS (liquid chromatography coupled with electrospray-ionization quadrupole time-of-flight mass spectrometry) [2,3,4]. Mërtiri et al. reported that the extracts of juniper berries have antioxidant and antibacterial activity [5]. Ivanova et al. found that juniper leaf extracts contain a great diversity of lignans (podophyllotoxin, deoxypodophyllotoxin) [6].

*J. communis* is the species in this genus that is widely used in wine and gin production. The bioactivities of folk medicinal plants in this genus include spasmolytic, wound healing, antioxidant, anti-inflammatory, antimicrobial, hypoglycemic, neuroprotective, antidiarrheal, analgesic, antipyretic, hypotensive, cardioprotective, anticancer, and antileishmanial effects, etc. [6,7]. Alhayyani et al. reported that *J. procera* leaves’ methanolic extract has anticancer activity [7]. Mediavilla et al. analyzed the activities of the essential oil steam-distilled from the leaves of *J. communis* following the Cascade Principle of biomass, which were crushed and steam-distilled. And the coarse fraction obtained from the separation of distilled juniper residual biomass, as pyrolysed in the pilot scale and separated into fractions to produce biochar and absorbents for the pet industry [6]. Meringolo et al. reported that essential oils and Extracts of *J. macrocarpa* Sm. and *J. oxycedrus* possess the antioxidant and anti-proliferative activities [8]. Raasmaja et al. found the water extract of *Juniperus communis* L. induces cell death and sensitizes cancer cells to cytostatic drugs through p53 and PI3K/Akt Pathways [9]. Raina et al. reported that the plant *Juniperus communis* L. is rich in aromatic oils, invert sugars, resins, catechin, organic acid, terpenic acids, leucoanthocyanidin, alkaloids, flavonoids, tannins, gums, lignins, wax, etc., and has been used for thousands of years as an herbal medicine for the treatment of diseases in human and animals [10].

Mosquitoes are common insects in daily life. Mosquitoes would transmit pathogens that cause serious, life-threatening diseases such as malaria, dengue, chikungunya, West Nile fever, yellow fever, and Zika [11,12,13,14]. Dengue (break-bone fever) is a disease caused by the dengue virus, and in 1907, the pathogen was found to be a virus, which comprises four serotypes (DENV-1-4) with both increasing dengue incidence, with an estimated 390 million cases reported annually, and case fatality [15,16]. According to the World Health Organization (WHO), approximately half of the global population is currently at risk of dengue, with an estimated 100 to 400 million infections occurring annually, which is the viral infection transmitted to humans through the bite of infected female vector mosquitoes, mainly by *Aedes aegypti* and *Ae. albopictus* [17]. Synthetic insecticides (such as permethrin, cypermethrin, and deltamethrin), organophosphates (like malathion), and carbamates have been used to reduce the transmission of mosquito-borne diseases for decades [17,18]. Currently, the extensive use of synthetic compounds has increased substantially, making mosquito-borne disease elimination and prevention more difficult over the years due to insecticide resistance in mosquitoes [19,20]. Mosquito-borne diseases are currently considered important threats, as increasing drug resistance to synthetic larvicides leads to more mosquito-borne diseases. Many researchers investigated the new alternative drug of synthetic larvicides from plant natural products with great interest [21,22,23,24,25,26]. Karunamoorthi et al. reported that the essential oil of *Juniperus procera* (*J. procera*) (Cupressaceae) possessed the larvicidal activity against late third instar larvae of Anopheles arabiensis (*An. arabiensis*) Patton, which is the principal malaria vector in Ethiopia [27].

The aims of this study are to analyze its chemical composition and to evaluate the mosquito larvicidal activity of leaf essential oil and its constituents from *J. chinensis* var. *kaizuka* against dengue vector mosquitoes, *Aedes aegypti* and *Ae. albopictus*.

## 2. Results and Discussion

### 2.1. Chemical Constituents of Juniperus chinensis var. kaizuka Leaf Essential Oil

The yield of leaf essential oil of *J. chinensis* var. *kaizuka* was 0.82 ± 0.02% after 6 h hydrodistillation. The constituents of *A. dammara* leaf essential oil were analyzed by using GC-MS, and 21 constituents were found in the gas chromatogram. According to Table 1, the constituents, comprising 92.67 ± 0.41% the total composition, were monoterpenes (50.19 ± 2.10%), oxygenated monoterpenes (25.05 ± 0.91%), sesquiterpene hydrocarbon (0.49 ± 0.02%), diterpene hydrocarbon (13.64 ± 1.46%), and oxygenated sesquiterpene (16.94 ± 2.52%). Most of the major constituents were oxygenated monoterpenes, containing bornyl acetate (23.71 ± 0.73%) and β-Elemol (14.99 ± 1.73%).

Sowndhararajan et al. analyzed the composition of essential oils from the needles, twigs, and berries of *Juniperus chinensis* L. in Korea. The major were bornyl acetate (2.85–20.70%), sabinene (10.23–18.13%), α-pinene (5.80–16.26), terpinen-4-ol (5.98–31.10), limonene (3.98–6.96%), β-pinene (3.05–4.39%), γ-terpinene (2.24–8.36%), α-elemol (1.74–4.77%) and α-cadinol (2.49–3.39%) [2,20]. Constituents of *J. chinensis* var. *kaizuka* leaf essential oil in Taiwan were similar to those of *J. chinensis* L. in Korea. Differences between two studies of chemical constituents of leaf essential oil may be due to the solar energy, maturity of leaf, collected region, environmental temperature, relative humidity, season, etc. [28].

**Table 1:** Constituents of essential oil from *J. chinensis* var. *kaizuka* leaves.

RT (min)	Compound	Formula	KI ^a^	rKI ^b^	Relative Content (%)
7.06	α-Pinene	C_10_H_16_	935	939	0.98 ± 0.04
7.62	Camphene	C_10_H_16_	952	954	0.93 ± 0.04
8.45	Sabinene	C_10_H_16_	974	975	3.54 ± 0.13
9.11	β-Myrcene	C_10_H_16_	990	990	8.11 ± 0.25
10.16	α-Terpinene	C_10_H_16_	1017	1017	0.73 ± 0.03
10.77	Limonene	C_10_H_16_	1033	1029	33.33 ± 1.14
11.90	γ-Terpinene	C_10_H_16_	1059	1059	1.12 ± 0.04
13.08	Terpinolene	C_10_H_16_	1085	1088	1.44 ± 0.05
13.74	Linalool	C_10_H_18_O	1098	1096	0.11 ± 0.02
17.34	Terpinen-4-ol	C_10_H_18_O	1179	1177	1.23 ± 0.02
22.14	Bornyl acetate	C_12_H_20_O_2_	1284	1285	23.71 ± 0.73
26.28	β-Elemene	C_15_H_24_	1388	1390	0.49 ± 0.01
30.31	Cubebol	C_15_H_26_O	1515	1515	1.41 ± 0.03
31.20	β-Elemol	C_15_H_26_O	1549	1549	14.99 ± 1.73
33.33	γ-Eudesmol	C_15_H_26_O	1630	1632	0.54 ± 0.38
Monoterpenes					50.19 ± 2.10
Oxygenated monoterpenes					25.05 ± 0.91
Sesquiterpenes					0.49 ± 0.02
Oxygenated sesquiterpenes					16.94 ± 2.52
Total identified					92.67 ± 0.41

^a^ KI: Kovats index relative to *n*-alkanes (C7–C30) on a DB-5MS column; ^b^ rKI: reference Kovats index from research [29].

### 2.2. Brine Shrimp Lethality Activity of Juniperus chinensis var. kaizuka Leaf Essential Oil

**Figure 1:**
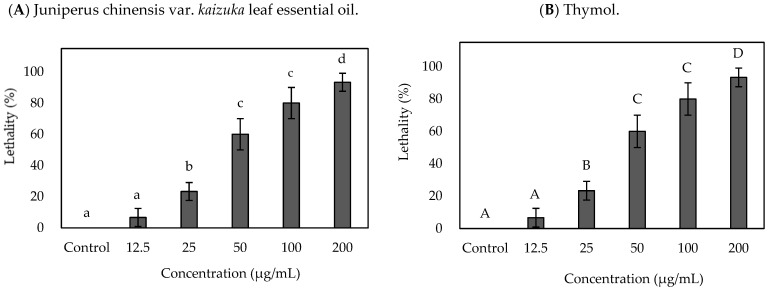
Brine shrimp lethality activity of *Juniperus chinensis* var. *kaizuka* leaf essential oil and thymol. (**A**) *Juniperus chinensis* var. *kaizuka* leaf essential oil; (**B**) Thymol. Different letters in the Figure are referred to as the significant difference at the level of *p* < 0.05 according to Scheffe’s test.

Figure 1 shows the lethal activity of *Juniperus* leaves’ essential oil and thymol against the brine shrimp, respectively. A dose-dependent relationship is observed in lethal activity to brine shrimp with *J. chinensis* var. *kaizuka* leaves’ essential oil. When 50 μg/mL of essential oil is applied to brine shrimp, the mortality rate is higher than 50%. The lethal concentration value of 50% mortality, LC_50_ value, is 49.89 μg/mL of essential oil to brine shrimp. The bark of *Duguetia lanceolata* vulgaris, an Annonaceae plant, contains main constituents such as β-Elemene, β-Selinene, and Caryophyllene oxide with steam distillation extraction. After 4 h of extraction time, the LC_50_ value of this essential oil is 60.7 μg/mL to brine shrimp [30]. These studies indicate good lethal activities against brine shrimp with *Juniperus* leaves essential oil. Previous studies consider that the good lethal activity to brine shrimp corresponded to the larvicidal activity of insects and antitumor activity [30,31,32,33]. Further study will evaluate the potential of *Juniperus* leaves’ essential oil. Figure 1B shows the positive control group of Thymol; the LC_50_ value of this essential oil is 15.97 μg/mL. Thymol is generally recognized as safe (GRAS) by the United States Food and Drug Administration, USFDA. Thymol also affects cancer cell line activity. Blažíčková et al. (2022) found the LC_50_ values of the human colorectal cancer cell line (HCT-116) are 65 μg/mL with Thymol [32,34,35]. The brine shrimp toxicity test has been used to evaluate the extracts for larvicidal activity against insects and potential cytotoxic activity of cancer cells [32,36,37,38]. After brine shrimp nauplii were treated with different specimens for 24 h, the number of dead nauplii was counted and converted to lethality. Figure 1 shows the lethal activity of *J. chinensis* var. *kaizuka* leaves’ essential oil and Thymol. Both the treatments of leaf essential oil reached 100% lethality after 24 h incubation at the concentrations of 100 and 200 μg/mL. At the concentrations of 50 μg/mL and 25 μg/mL, leaf essential oil caused 63.3 ± 5.77% and 27.5 ± 9.57% lethality of brine shrimp, respectively. A dose-dependent relationship is observed in lethal activity to brine shrimp with *Juniperus* leaves essential oil. When 50 μg/mL of essential oil is applied to brine shrimp, the mortality rate is higher than 50%. The lethal concentration value of 50% mortality, LC_50_ value, is 49.89 μg/mL of essential oil to brine shrimp. Niksic et al. (2021) analyzed the *Thymus vulgaris* essential oil using steam distillation and evaluated brine shrimp lethal activity; results show that the LC_50_ value is 60.38 μg/mL [30]. The bark of *Duguetia lanceolata* vulgaris, an Annonaceae plant, contains main constituents such as β-Elemene, β-Selinene, and Caryophyllene oxide. With steam distillation extraction, the LC_50_ value of this essential oil is 60.7 μg/mL to brine shrimp [34]. These studies indicate good lethal activities against brine shrimp with *Juniperus* leaves essential oil. Previous studies consider that the good lethal activity to brine shrimp relates to antitumor activity [31,38,39]. Imran et al. (2021) evaluate the anti-parasitic, insecticidal, cytotoxic and anti-alzheimer potential of *Ajuga bracteosa* Wallich ex Bentham leaf extracts; results revealed that natural prod-ucts with significant cytotoxic potential against Artemia salina are worth further ex-ploration its bioactivities [40].

After brine shrimp nauplii were treated with different specimens for 24 h, the number of dead nauplii was counted and converted to lethality; the result is shown in Figure 1. Both the treatments of leaf essential oil reached 100% lethality after 24 h incubation at the concentrations of 100 and 200 μg/mL. At concentrations of 50 μg/mL and 25 μg/mL, leaf essential oil caused 63.3 ± 5.77% and 27.5 ± 9.57% lethality of brine shrimp, respectively.

### 2.3. Brine Shrimp Lethality Activity of J. chinensis var. kaizuka Leaf Essential Oil Against Dengue Vector Mosquitoes

After brine shrimp nauplii were treated with different specimens for 24 h, the number of dead nauplii was counted and converted to lethality; the result is shown in Figure 1. Both the treatments of leaf essential oil reached 100% lethality after 24 h incubation at the concentrations of 100 and 200 μg/mL. At the concentrations of 50 μg/mL and 25 μg/mL, leaf essential oil caused 63.3 ± 5.77% and 27.5 ± 9.57% lethality of brine shrimp, respectively. Table 2: Regarding the effective lethal concentration (LC_50_ and LC_90_) of *J. chinensis* var. *kaizuka* leaf essential oil and thymol, positive control, against brine shrimp after 24 h of treatment. LC_50_ and LC_90_ values of leaf essential oil were 43.06 ± 1.97 μg/mL and 88.20 ± 2.92 μg/mL, respectively, after 24 h. The toxicity of *J. chinensis* var. *kaizuka* leaf essential oil, with LC_50_ values below 100 μg/mL, can be categorized into toxic/highly toxic levels [41,42]. LC_50_ and LC_90_ values of thymol were 8.43 ± 1.46 and 15.99 ± 1.75 μg/mL, respectively, after 24 h. Result of LC_90_ values that were consistent with the findings of Niksic et al. reported in the 95% confidence interval of LC_50_ of thymol were 5.20–56.29 μg/mL [30]. The bark of *Duguetia lanceolata* vulgaris, an Annonaceae plant, contains main constituents such as β-elemene, β-selinene, and caryophyllene oxide with steam distillation extraction. The LC_50_ value of this essential oil is 60.7 μg/mL to brine shrimp [30].

### 2.4. Mosquito Larvicidal Activity of J. chinensis var. kaizuka Leaf Essential Oil and Its Constituents

Extensive uses of synthetic insecticides cause global damage to human health and the ecosystem [12,17,18,19,20]. Synthetic insecticides also pollute the aquatic environment, which is a hazardous worldwide problem, and induce long-term harmful effects on living aquatic organisms [19]. More bio-insecticides and bio-larvicides are found in natural products [19]. Table 3 presents the Effective lethal concentrations of *J. chinensis* var. *kaizuka* leaf essential oil and rotenone, a broad-spectrum natural insecticide and pesticide [43,44], against *Ae. aegypti* larvae.

*Ae. aegypti* transmits the pathogens that cause the Zika fever, dengue fever, yellow fever, and chikungunya [18,19]. Suppressing the proliferative ability is one of the solutions to reduce the transmission of mosquito-related diseases. The results displayed that leaf essential oil could be effective against fourth-instar *Ae. aegypti* larvae after 48 h incubation. The treatment with leaf essential oil caused 40 ± 8.16% mortality at 24 h and 82.50 ± 5.0% mortality at 48 h at a concentration of 200 μg/mL. As for the positive control, rotenone possessed *Ae. aegypti* larvicidal activity when the concentration was above 3.75 μg/mL and 0.938 μg/mL at 24 h and 48 h, respectively. LC_50_ and LC_90_ of specimens are shown in Table 4. LC_50_ and LC_90_ values of leaf essential oil were 155.04 ± 8.44 μg/mL and 207.07 ± 6.98 μg/mL, respectively, after 48 h.

Govindarajan et al. reported that δ-cadinene is one of the major constituents of *Kadsura heteroclita* essential oil, and δ-cadinene is effective against *Ae. aegypti* larvae [45]. The LC_50_ of δ-cadinene was 9.03 μg/mL. *J. chinensis* var. *kaizuka* leaf essential oil presented similar or higher larvicidal activity than *Santalum album* essential oil and the methanolic extracts of *Blumea mollis*, *Tagetes erecta*, and *Lantana camera* [20,32]. The LC_50_ and LC_90_ of *S. Santalum album* essential oil were 250.64 μg/mL and 709.06 μg/mL, respectively [20]. The LC_50_ of the methanolic extracts of *Blumea mollis*, *Tagetes erecta*, and *Lantana camera* were 273.68, 275.80, and 445.52 μg/mL, respectively [19].

Table 4 shows the effective lethal concentrations of *Ae. albopictus* larvae treated with leaf essential oil and its compounds. The order of mosquito larvicidal activity of the main constituents of leaf essential oil is Limonene > β-Myrcene > Sabinene > Bornyl acetate. Among the essential oil components, limonene exhibited the strongest larvicidal activity against *Aedes aegypti*. After 48 h of treatment, the LC_50_ and LC_90_ values for limonene were 36.40 μg/mL and 70.41 μg/mL, respectively, indicating its potential as an effective larvicide. After 48 h of treatment, the LC_50_ and LC_90_ values for limonene were 36.40 μg/mL and 70.41 μg/mL, respectively. The LC_50_ value of sabinene against *Aedes aegypti* larvae was also below 100 μg/mL. After 48 h of exposure, the LC_50_ and LC_90_ values for sabinene were 70.77 μg/mL and 70.41 μg/mL, respectively. Bornyl acetate showed lower larvicidal efficacy compared to other essential oil components, with LC_50_ and LC_90_ values of 102.43 μg/mL and 181.00 μg/mL, respectively, after 48 h of treatment.

Table 5 shows the lethal activity of compounds of *J. chinensis* var. *kaizuka* leaf essential oil. The order of mosquito larvicidal activity of the main constituents of leaf essential oil is Limonene > β-Myrcene > Sabinene > Bornyl acetate. Among them, limonene also performed the best lethal activity against *Ae. albopictus* larvae than that of the leaves’ essential oil. LC_50_ value for a processing period of 24 h for leaves’ essential oil is 46.74 μg/mL, and that for Limonene is 24.12 μg/mL. LC_90_ value of Limonene after 48 h processing time is 41.80 μg/mL. All of them are below 50 μg/mL. Following those results, after 48 h processing time, the LC_50_ values of Sabinene and β-Myrcene to *Ae. albopictus* larvae are 58.63 μg/mL and 53.52 μg/mL, and the LC_90_ of those are 114.67 μg/mL and 94.25 μg/mL. The activity of bornyl acetate is lower than that of other compounds. After 48 h of treatment, the LC50 and LC90 values are 146.57 μg/mL and 193.39 μg/mL, respectively. Except for Bornyl acetate, *J. chinensis* var. *kaizuka* leaves essential oil and its compounds (except bornyl acetate) all have good larvicidal activity against *albopictus* larvae, showing potential to be natural pesticides to kill *Ae. albopictus* larvae.

**Figure 2:**
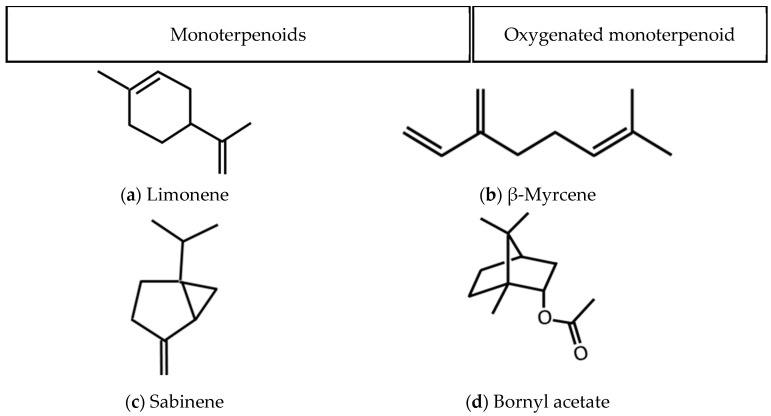
Chemical structures of major compounds of *J. chinensis* var. *kaizuka* leaf essential oil.

Figure 2 shows the chemical structures of major compounds of *J. chinensis* var. *kaizuka* leaf essential oil. Among the major compounds of leaf oil, limonene, β-myrcene, and sabinene showed the best mosquito larvicidal effect. These active compounds all contain the isopropenyl group. Results revealed that monoterpenoids with an isopropenyl group may exhibit superior mosquito larvicidal activity.

## 3. Materials and Methods

### 3.1. Plant Material

The fresh leaves of *Juniperus chinensis* var. *kaizuka* were collected from the campus (25.017° N, 121.540° E) of National Taiwan University, Taipei, Taiwan, in April 2022. The species was identified, and a voucher specimen (JC0423) was deposited in the Lab of Chemical Utilization of Biomaterials, School of Forestry and Resource Conservation, National Taiwan University.

### 3.2. Hydrodistillation of Essential Oil

The freshly collected leaves of *J. chinensis* var. *kaizuka* were hydrodistilled for 6 h using the Clevenger-type apparatus. Leaf essential oil was stored in airtight containers for further investigation [46,47,48].

### 3.3. Gas Chromatography-Mass Spectrometry (GC-MS) Analysis

To investigate the chemical composition of the essential oil, the analysis of leaf oil was carried out on a Trace GC Ultra (Thermo Fisher Scientific, Waltham, MA, USA) equipped with a DB-5 MS column (Crossbond 5% methylpolysiloxane, 30.0 m length × 0.25 mm diameter, thickness 0.25 μm; Agilent Technologies, Santa Clara, CA, USA). The oven temperature started from 60 °C for 3 min, programmed at 3 °C min^−1^ to 120 °C, 5 °C min^−1^ to 240 °C for 3 min. The injector temperature was held at 250 °C; injection size 1 μL neat; split ratio 10:1. The carrier gas was helium; 1.0 mL/min flow rate; ion source temperature 250 °C; mass range 50–650 amu. The constituents of essential oil were characterized by the National Institute of Standards and Technology (NIST) V.2.0 and Wiley 7.0 GC-MS libraries and Kovats indices in the reference [29]. Kovats’ indices of the constituents are determined by retention times of *n*-alkanes (C7–C30) on the DB-5MS column. The relative contents of constituents were determined by the peak area of the spectrum [28,45,49,50,51].

### 3.4. Mosquito Larvicidal Assay

Fourth-instar larvae of *Aedes aegypti* were obtained from Prof. Tsai’s lab at the Department of Public Health, National Taiwan University. The mosquito larvicidal assay was performed at room temperature. Ten larvae, 19.80 mL ddH_2_O, and 200 μL specimen were added to an experimental vial and incubated for 48 h. The experiment was performed in quadruplicate. Mortality was recorded at 24 h and 48 h, respectively. Median lethal concentration (LC_50_) and LC_90_ were calculated based on the mortality of each treatment. Specimens, leaf essential oil, and rotenone are dissolved in DMSO; rotenone (natural insecticide) is the positive control [31,38,52].

### 3.5. Brine Shrimp Lethality Assay

Brine shrimp lethality assay was performed according to the related studies [18]. The eggs of brine shrimp (*Artemia salina* Leach) were hatched in a segregated plastic tray filled with sea salt water (35 g/L). The time for hatching brine shrimp nauplii required approximately 48 h. To perform BST, ten nauplii were placed in an experimental vial. Then, 50 μL of leaf essential oil and 4.95 mL seawater were added to each vial. Leaf essential oil was dissolved in DMSO. The positive control is thymol. The experiment was performed in quadruplicate. The vials containing sea salt water and specimens were incubated for 24 h at room temperature. After the incubation, the number of dead nauplii was counted under a stereomicroscope (Hamlet SEM-H, Taipei, Taiwan) and converted into lethality (%), LC_50_, and LC_90_ [30,32,33].

### 3.6. Statistical Analysis

The Statistical Analysis and Research of data obtained in the research was analyzed by SPSS (Statistical Product and Service Solutions) (Chicago, IL, USA) Version 16 with Scheffe’s multiple comparison test, a post hoc multiple comparison method. The significance level was set to 5%. (α = 0.05).

## 4. Conclusions

The chemical constituents of *J. chinensis* var. *kaizuka* leaf essential oil. The Chemical composition of JUNIPERUS was analyzed by gas chromatography-mass spectrometry, and they were mainly monoterpenoids. Its major compounds were limonene (33.33%), bornyl acetate (23.71%), β-elemol (14.99%), β-myrcene (8.11%), and sabinene (3.54%). In the mosquito larvicidal activity assay, *J. chinensis* var. *kaizuka* and limonene demonstrate significant mosquito larvicidal activity against fourth-instar larvae of *Aedes aegypti* and *Ae. albopictus*, *J. chinensis* var. *kaizuka* leaf essential oil also presented excellent lethality against Artemia salina (brine shrimp). Sabinene showed the closest activity effect to essential oil; both LC_50_ values were lower than 50 μg/mL, and LC_90_ values were lower than 200 μg/mL. *J. chinensis* var. *kaizuka* leaf essential oil, which is found in rich larvicidal monoterpenoids, may be applied to the mosquito larvicidal activity for mosquito control against Dengue Vector Mosquitoes larvae, *Aedes aegypti* and *Ae. albopictus*. Results revealed that monoterpenoids with an isopropenyl group may exhibit superior mosquito larvicidal activity.

## Figures and Tables

**Table 2:** Effective lethal concentration of *Juniperus chinensis* var. *kaizuka* leaf essential oil against brine shrimp.

Specimen	LC_50_ (μg/mL)	LC_90_ (μg/mL)
Leaf essential oil	43.06 ± 1.97 ^b^	88.20 ± 2.92 ^B^
Thymol *	8.43 ± 1.46 ^a^	15.99 ± 1.75 ^A^

*: Positive control; Different letters (a,b; A,B) in the Table are referred to as significant difference at the level of *p* < 0.05 according to Scheffe’s test.

**Table 3:** Effective lethal concentrations of *Juniperus chinensis* var. *kaizuka* leaf essential oil against *A. aegypti* larvae.

Specimen	24 h		48 h	
	LC_50_	LC_90_	LC_50_	LC_90_
Leaf essential oil	213.52 ± 11.23 ^c^	272.70 ± 22.18 ^d^	155.04 ± 8.44 ^b^	207.07 ± 6.98 ^c^
Rotenone *	4.89 ± 0.66 ^a^	-	1.61 ± 0.15 ^a^	5.32 ± 0.61 ^a^

Unit: μg/mL; *: positive control; -: >20 μg/mL; Different letters (a–d) refer to significant difference at the level of *p* < 0.05 according to Scheffe’s test.

**Table 4:** Effective lethal concentrations to *Ae. aegypti* with leaf essential oil and its compounds.

Specimen	LC_50_		LC_90_	
	24 h	48 h	24 h	48 h
Leaf oil	27.51 ± 1.25 ^bc^	25.46 ± 0.86 ^bc^	44.44 ± 4.95 ^B^	44.40 ± 1.98 ^B^
Limonene	35.72 ± 3.91 ^cd^	28.73 ± 8.95 ^c^	76.01 ± 2.38 ^C^	74.07 ± 2.10 ^C^
Sabinene	55.62 ± 10.65 ^e^	49.94 ± 17.36 ^de^	137.99 ± 26.21 ^D^	131.61 ± 30.50 ^D^
β-Myrcene	36.10 ± 1.41 ^d^	36.40 ± 1.53 ^d^	70.77 ± 2.51 ^C^	70.41 ± 2.34 ^C^
Bornyl acetate	111.63 ± 20.85 ^f^	102.43 ± 8.66 ^f^	183.45 ± 6.06 ^A^	181.00 ± 4.98 ^A^
Rotenone *	21.11 ± 5.77 ^bc^	10.78 ± 2.71 ^ab^	- **	-

Unit of essential oil and compounds is μg/mL; Different letters in the Table represent the significantly different at the level of *p* < 0.05 according to the Scheffe test. *: Positive control. **: No effect (>200 μg/mL).

**Table 5:** Effective lethal concentrations to *Ae. albopictus* with leaf essential oil and its compounds.

Specimen	LC_50_		LC_90_	
	24 h	48 h	24 h	48 h
Leaf oil	46.74 ± 10.62 ^b^	46.64 ± 10.79 ^b^	65.94 ± 8.85 ^B^	66.36 ± 8.01 ^B^
Sabinene	59.57 ± 3.97 ^c^	58.63 ± 3.53 ^c^	113.05 ± 9.12 ^D^	114.67 ± 12.08 ^D^
β-Myrcene	57.13 ± 5.97 ^bc^	53.52 ± 7.46 ^bc^	105.43 ± 14.33 ^CE^	94.25 ± 14.51 ^C^
Limonene	24.12 ± 0.65 ^a^	23.86 ± 1.15 ^a^	42.30 ± 1.67 ^A^	41.80 ± 2.55 ^A^
Bornyl acetate	150.02 ± 12.77 ^d^	146.57 ± 11.20 ^e^	194.09 ± 2.94 ^E^	193.39 ± 3.45 ^E^
Rotenone *	- **	-	-	-

Unit of essential oil and compounds is μg/mL; Different letters in the Table represent the significantly different at the level of *p* < 0.05 according to the Scheffe test. *: Positive control. **: No effect (>50 μg/mL).

## Data Availability

The data are available from the corresponding author on reasonable request.

## References

[1] Belov T., Terenzhev D., Bushmeleva K.N., Davydova L., Burkin K., Fitsev I., Gatiyatullina A., Egorova A., Nikitin E. (2023). comparative analysis of chemical profile and biological activity of *Juniperus communis* L. Berry extracts. Plants.

[2] Höferl M., Stoilova I., Schmidt E., Wanner J., Jirovetz L., Trifonova D., Krastev L., Krastanov A. (2014). Chemical composition and antioxidant properties of Juniper berry (*Juniperus communis* L.) Essential Oil. Action of the essential oil on the antioxidant Protection of Saccharomyces cerevisiae model organism. Antioxidants.

[3] Hrytsyna M., Salamon I., Peleno R., Vargova V. (2024). Identification and analysis of the content of biologically active substances of juniper cone berries and their antioxidant activity. Horticulturae.

[4] Tang J., Dunshea F.R., Suleria H.A.R. (2019). LC-ESI-QTOF/MS Characterization of phenolic compounds from medicinal plants (Hops and Juniper Berries) and their antioxidant activity. Foods.

[5] Mërtiri I., Păcularu-Burada B., Stănciuc N. (2024). Phytochemical characterization and antibacterial activity of Albanian *Juniperus communis* and *Juniperus oxycedrus* berries and needle leaves extracts. Antioxidants.

[6] Ivanova D.I., Nedialkov P.T., Tashev A.N., Olech M., Nowak R., Ilieva Y.E., Kokanova-Nedialkova Z.K., Atanasova T.N., Angelov G., Najdenski H.M. (2021). Junipers of Various Origins as Potential Sources of the Anticancer Drug Precursor Podophyllotoxin. Molecules.

[7] Alhayyani S., Akhdhar A., Asseri A.H., Mohammed A.M.A., Hussien M.A., Roselin L.S., Hosawi S., AlAbbasi F., Alharbi K.H., Baty R.S. (2023). Potential Anticancer Activity of *Juniperus procera* and Molecular Docking Models of Active Proteins in Cancer Cells. Molecules.

[8] Meringolo L., Bonesi M., Sicari V., Rovito S., Passalacqua N.G., Loizzo M.R., Tundis R. (2022). Essential Oils and Extracts of Juniperus macrocarpa Sm. and Juniperus oxycedrus L.: Comparative Phytochemical Composition and Anti-Proliferative and Antioxidant Activities. Plants.

[9] Raasmaja A., Stenius U., Ghalali A. (2019). The water extract of *Juniperus communis* l. induces cell death and sensitizes cancer cells to cytostatic Drugs through p53 and PI3K/Akt Pathways. Int. J. Mol. Sci..

[10] Raina R., Verma P.K., Peshin R., Kour H. (2019). Potential of *Juniperus communis* L as a nutraceutical in human and veterinary medicine. Heliyon.

[11] Hribar L.J., Boehmler M.B., Murray H.L., Pruszynski C.A., Leal A.L. (2022). Mosquito Surveillance and Insecticide Resistance Monitoring Conducted by the Florida Keys Mosquito Control District, Monroe County, Florida, USA. Insects.

[12] Lees R.S., Fornadel C., Snetselaar J., Wagman J., Spiers A. (2023). Insecticides for Mosquito Control: Improving and Validating Methods to Strengthen the Evidence Base. Insects.

[13] Borovsky D. (2003). Biosynthesis and control of mosquito gut proteases. IUBMB Life.

[14] Chen W.J. (2018). Dengue outbreaks and the geographic distribution of dengue vectors in Taiwan: A 20-year epidemiological analysis. Biomed. J..

[15] See K.C. (2025). Dengue Vaccination: A Practical Guide for Clinicians. Vaccines.

[16] Anumanthan G., Sahay B., Mergia A. (2025). Current Dengue virus vaccine developments and future directions. Viruses.

[17] World Health Organization Dengue and Severe Dengue. https://www.who.int/news-room/fact-sheets/detail/dengue-and-severe-dengue.

[18] Osanloo M., Sedaghat M.M., Sanei-Dehkordi A., Amani A. (2019). Plant-Derived Essential Oils; Their Larvicidal Properties and Potential Application for Control of Mosquito-Borne Diseases. Galen Med J..

[19] Senthil-Nathan S. (2020). A Review of resistance mechanisms of synthetic insecticides and botanicals, phytochemicals, and essential oils as alternative larvicidal agents against mosquitoes. Front. Physiol..

[20] Malijan R.P.B., Angeles J.R., Apilado A.M.A., Ammugauan M.A.T., Salazar F.V. (2024). Insecticide Resistance in Aedes aegypti from the National Capital Region of the Philippines. Insects.

[21] Farag M.R., Alagawany M., Bilal R.M., Gewida A.G.A., Dhama K., Abdel-Latif H.M.R., Amer M.S., Rivero-Perez N., Zaragoza-Bastida A., Binnaser Y.S. (2021). An overview on the potential hazards of pyrethroid insecticides in fish, with Special emphasis on cypermethrin toxicity. Animals.

[22] Ahamad A., Kumar J. (2023). Pyrethroid pesticides: An overview on classification, toxicological assessment and monitoring. J. Hazard. Mater. Adv..

[23] Frezza C., Venditti A., De Vita D., Toniolo C., Franceschin M., Ventrone A., Tomassini L., Foddai S., Guiso M., Nicoletti M. (2020). Phytochemistry, Chemotaxonomy, and Biological Activities of the Araucariaceae Family-A Review. Plants.

[24] Carroll J.F., Tabanca N., Kramer M., Elejalde N.M., Wedge D.E., Bernier U.R., Coy M., Becnel J.J., Demirci B., Başer K.H. (2011). Essential oils of Cupressus funebris, *Juniperus communis*, and *J. chinensis* (Cupressaceae) as repellents against ticks (Acari: Ixodidae) and mosquitoes (Diptera: Culicidae) and as toxicants against mosquitoes. J. Vector Ecol..

[25] Luz T.R.S.A., de Mesquita L.S.S., Amaral F.M.M.D., Coutinho D.F. (2020). Essential oils and their chemical constituents against *Aedes aegypti* L. (Diptera: Culicidae) larvae. Acta Trop..

[26] Araujo M.O., Perez-Castillo Y., Oliveira L.H.G., Nunes F.C., de Sousa D.P. (2021). Larvicidal activity of cinnamic acid derivatives: Investigating alternative products for *Aedes aegypti* L. control. Molecules.

[27] Karunamoorthi K., Girmay A., Fekadu S. (2014). Larvicidal efficacy of Ethiopian ethnomedicinal plant *Juniperus procera* essential oil against Afrotropical malaria vector *Anopheles arabiensis* (Diptera: Culicidae). Asian Pac. J. Trop. Biomed..

[28] Ho Y.T., Liu I.H., Chang S.T., Wang S.Y., Chang H.T. (2023). *In vitro* and *in vivo* antimelanogenesis effects of leaf essential oil from *Agathis dammara*. Pharmaceutics.

[29] Adams R.P. (2007). Identification of Essential Oil Components by Gas Chromatography/Mass Spectrometry.

[30] Niksic H., Becic F., Koric E., Gusic I., Omeragic E., Muratovic S., Miladinovic B., Duric K. (2021). Cytotoxicity screening of *Thymus vulgaris* L. essential oil in brine shrimp nauplii and cancer cell lines. Sci. Rep..

[31] Meyer B.N., Ferrigni N.R., Putnam J.E., Jacobsen L.B., Nichols D.E., McLaughlin J.L. (1982). Brine shrimp: A convenient general bioassay for active plant constituents. Planta Med..

[32] Sousa O.V., Del-Vechio-Vieira G., Alves M.S., Araújo A.A., Pinto M.A., Amaral M.P., Rodarte M.P., Kaplan M.A. (2012). Chemical composition and biological activities of the essential oils from *Duguetia lanceolata* St. Hil. barks. Molecules.

[33] Bıtgen N., Onder G.O., Baran M., Yay A. (2023). Cytotoxicity screening of *Thymus vulgaris* L. in breast cancer: In vitro study. Toxicol. Res..

[34] Waghulde S., Kale M.K., Patil V.R. (2019). Brine shrimp lethality assay of the aqueous and ethanolic extracts of the selected species of medicinal plants. Proceedings.

[35] Blažíčková M., Blaško J., Kubinec R., Kozics K. (2022). Newly synthesized thymol derivative and its effect on colorectal cancer cells. Molecules.

[36] Moola S., Orchard A., van Vuuren S. (2023). The Antimicrobial and toxicity influence of six carrier oils on essential oil compounds. Molecules.

[37] Reddy P.G., Domb A.J. (2023). Bioactive phenolate salts: Thymol salts. Chem. Med. Chem..

[38] Sowndhararajan K., Seo M., Kim S. (2016). Comparative analysis of the composition of essential oils from the needles, twigs and berries of *Juniperus chinensis* L. in Korea. J. Appl. Pharm. Sci..

[39] Jayaraman M., Senthilkumar A., Venkatesalu V. (2015). Evaluation of some aromatic plant extracts for mosquito larvicidal potential against *Culex quinquefasciatus*, *Aedes aegypti*, and *Anopheles stephensi*. Parasitol. Res..

[40] Imran M., Jan H., Faisal S., Ali Shah S., Shah S., Naeem Khan M., Taj Akbar M., Rizwan M., Jan F., Syed S. (2021). In vitro examination of anti-parasitic, anti-Alzheimer, insecticidal and cytotoxic potential of *Ajuga bracteosa* Wallich leaves extracts. Saudi J. Biol. Sci..

[41] Nondo R.S., Mbwambo Z.H., Kidukuli A.W., Innocent E.M., Mihale M.J., Erasto P., Moshi M.J. (2011). Larvicidal, antimicrobial and brine shrimp activities of extracts from *Cissampelos mucronata* and *Tephrosia villosa* from coast region, Tanzania. BMC Complement. Altern. Med..

[42] Moshi M.J., Innocent E., Magadula J.J., Otieno D.F., Weisheit A., Mbabazi P.K., Nondo R.S. (2010). Brine shrimp toxicity of some plants used as traditional medicines in Kagera Region, north western Tanzania. Tanzan. J. Health Res..

[43] Sayono S., Anwar R., Sumanto D. (2020). Larvicidal activity of ethyl acetate extract of *Derris elliptica* Root against the Third-Instar Larvae of cypermethrin-resistant *Aedes aegypt*i Offspring. J. Arthropod-Borne Dis..

[44] Govindarajan M., Rajeswary M., Benelli G. (2016). δ-Cadinene, Calarene and δ-4-Carene from Kadsura heteroclita Essential Oil as Novel Larvicides Against Malaria, Dengue and Filariasis Mosquitoes. Comb. Chem. High Throughput Screen..

[45] Zhang Y., Lin X., Qian Y., Qin M., Zhang S., Wang L., Luo Y. (2025). Preparation and characterization of efficient and safe Rotenone solid nanodispersion by self-emulsifying technique. Nanomaterials.

[46] Chang H.T., Chang M.L., Chen Y.T., Chang S.T., Hsu F.L., Wu C.C., Ho C.K. (2021). Evaluation of motor coordination and anti-depressant activities of *Cinnamomum osmophloeum* ct. linalool leaf oil in rodent model. Molecules.

[47] Chen G.R., Chang M.L., Chang S.T., Ho Y.T., Chang H.T. (2022). Cytotoxicity and apoptosis induction of 6,7-dehydroroyleanone from *Taiwania cryptomerioides* bark essential oil in hepatocellular carcinoma cells. Pharmaceutics.

[48] Huang C.Y., Chang Y.Y., Chang S.T., Chang H.T. (2022). Xanthine oxidase inhibitory activity and chemical composition of *Pistacia chinensis* leaf essential oil. Pharmaceutics.

[49] Chang Y.-Y., Huang Y.-M., Chang H.-T. (2024). Using Headspace Gas Chromatography–Mass Spectrometry to Investigate the Volatile Terpenoids Released from the *Liquidambar formosana* Leaf and Its Essential Oil. Forests.

[50] Huang C.Y., Yeh T.F., Hsu F.L., Lin C.Y., Chang S.T., Chang H.-T. (2018). Xanthine oxidase inhibitory activity and thermostability of cinnamaldehyde-chemotype leaf oil of *Cinnamomum osmophloeum* microencapsulated with β-cyclodextrin. Molecules.

[51] Wu C.C., Huang S.L., Ko C.H., Chang H.T. (2022). Antifungal sesquiterpenoids from Michelia formosana leaf essential oil against wood-rotting fungi. Molecules.

[52] Chaita E., Lambrinidis G., Cheimonidi C., Agalou A., Beis D., Trougakos I., Mikros E., Skaltsounis A.L., Aligiannis N. (2017). Anti-melanogenic properties of greek plants. a novel depigmenting agent from Morus alba wood. Molecules.

